# Initiatives to enhance medical subspecialist training in Zambia: A cross-sectional analysis

**DOI:** 10.55320/mjz.49.1.36

**Published:** 2022-08-05

**Authors:** Violet Kayamba, Selestine Nzala, Moses Chikoti Simuyemba, Cosmas Zyambo, Emmanuel Musenge, Ruth Wahila, Victoria Mwiinga Kalusopa, Christabel Mwiinga, Linda Kampata-Olowski, Marjory Kabinga Makukula, Patricia Katowa-Mukwato, Elliot Kafumukache, Fastone Goma

**Affiliations:** 1 University of Zambia School of Medicine, Nationalist Road, P.O. Box 50110, Lusaka, Zambia; 2 Eden University, P.O. Box 37727, Barlastone Park, Lusaka, Zambia

**Keywords:** Medical specialist training, Zambia, Africa, Health professionals

## Abstract

**Introduction:**

There is a significant shortage of medical subspecialists in Zambia. The government of Zambia, through programmes at the Ministry of Health, spends considerable resources to send patients outside the country for subspecialist medical treatment. The objective of this analysis was to evaluate the current situation pertaining to medical subspecialty training at the University of Zambia School of Medicine (UNZASOM) and to illustrate the new programmes that are to be introduced.

**Methods:**

We collected data from formal desk reviews on the state of medical specialisation in Zambia, the UNZASOM graduation archives and patient referral records at the Ministry of Health (MoH). In addition, information on planned subspecialist programmes is presented.

**Results:**

From the first graduates in 1986up to 2019,UNZASOM produced 351medical specialists, 63 (18%) in Internal Medicine, 77 (22%) in Obstetrics &Gynaecology, 82 (23%) in Paediatrics&Child Health, 68 (19%) in General Surgery, 17 (5%) in Anaesthesia & Critical Care, 20 (6%) in Orthopaedics &Trauma and 8 (2%) in Urology. The remaining graduates were in Ophthalmology, Psychiatry, Infectious Diseases, Paediatric Surgery and Pathology contributing 1% each. To enhance medical subspecialist training at UNZASOM, new curricula for Breast Surgery, Urology, Glaucoma, Vitreo-retinalSurgery, Adult Gastroenterology, Forensic Pathology, Dermatology & Venereology, Ophthalmology, Gynaecological Oncology and Paediatric anaesthesia, Infectious Diseases, and Gastroenterology were developed. Since 2013, only 44% of patients requiring subspecialist treatment out of Zambia got assisted with the remainder still on the waiting list or having had bad outcomes.

**Conclusions:**

These programmes will provide an opportunity for accessible and affordable medical subspecialization training for Zambia and its neighbouring countries. With enhanced infrastructural support, the subspecialists will contribute toward enhanced healthcare provision and improvement in patient outcomes. They will also form a cohort of trainers to expand the space for quality training and skills building of specialists and subspecialists in the region and beyond.

## INTRODUCTION

The field of medicine is as expansive and multifaceted as the intricacies of the human body itself with various specialties and subspecialties.^1^ These specialties and subspecialties are recognized by medical professional bodies, organizations and regulatory authorities around the world. Each specialist area commands very specific skills accompanied by competencies and philosophies that make it unique. Low resource and relatively ‘young’ countries such as Zambia are lagging behind with a limited pool of medical specialists.^2^ There are several socio-economic and organizational reasons for this situation but the need for improvement is becoming increasingly obvious.

The medical landscape in Africa, including Zambia, is changing with a surge in non-communicable diseases (NCDs).^3^NCDs were traditionally overshadowed by a high burden of infectious diseases, which benefited from massive research and clinical care support from governmental and non-governmental local and international funders.^4^ Such investments, without doubt, greatly benefitted the Zambian health sector but it is becoming clear that survivors of these infectious diseases are now falling victim to NCDs as they live longer. In many cases, comprehensive care of NCDs demands multidisciplinary approaches requiring medical subspecialization. Over the past three decades, Africa has seen a massive evolution of postgraduate medical training^5^ and the University of Zambia School of Medicine (UNZASOM) is no exception.

UNZASOM was established in 1966 and anchored within the University Teaching Hospitals (UTH), the largest referral hospital in Zambia.^6^ Working in a mutually beneficial arrangement, UNZASOM and UTH have since then been producing clinicians who to this day make up the largest pool of medical doctors in Zambia. Medical specialty training demands human and material resources which are frequently scanty not only in Zambia but in many other African countries as well.^7^

Zambia has a high patient health care provider ratio^8,9^ resulting in great demand for doctors. The deficit for medical specialists is even worse and it is not unusual to find a single doctor running entire hospitals particularly outside big cities or provincial centres. For this reason, Zambia heavily relies on medical tourism, (travelling abroad for medical services), which in many cases is only available for a select few and significantly strains the already mergre resources.

The Strengthening Health Professional Workforce Education Programs for Improved Quality Health Care in Zambia (SHEPIZ) project, is a UNZASOM programme, under the Health Professional Education Partnership Initiative funded by the US National Institutes of Health. One of the aims of SHEPIZ is to foster the development of medical specialists leveraging local resources in conjunction with international partners. In this study, we report the process that was undertaken to collect information on medical specialization and subspecialization in Zambia, reviewing current achievements and highlighting innovations that have been instituted within UNZASOM.

## METHODS

A situation analysis was conducted of medical subspecialties in Zambia, a lower middle-income country. Data included in this manuscript was collected as follows:

### (1) Stakeholder engagement conducted under the Strengthening Health Professional Workforce Education Program for Improved Quality Health Care in Zambia (SHEPIZ) project.

We oragnised a week long workshop during which various stakeholders were invited to participate. We engaged consultants from UTH and UNZASOM departments of Pediatrics and Child Health, Internal Medicine, Surgery, Obstetrics and Gynaecology and Pathology to discuss various needs for medical subspecialisation in Zambia. In addition, we also invited some medical specialists in training (i.e Master of Medicine trainees). All medical consultants engaged had at least two years of practical experience. For guidance on preparation of training curricula, we engaged UNZASOM specialists from the department of Medical Education and Training. Information collected during the workshop from these key stakeholders helped identify areas that had enough capacity and resources to develop and run subspecialist programmes. In addition, information on the availability of international partner universities and specialists who would assist in the process of developing and/or running these programmes was sought.

### (2) Archived records for graduating students at the University of Zambia (UNZA)

Information was collected from UNZASOM archives of medical postgraduate students that graduated since inception of these programmes in 1983. Clinical postgraduate programmes at UNZASOM are run as full-time courses with the requirement of a relevant research project, resulting in a dissertation and submission of at least one peer reviewed publication. Full time and honorary UNZASOM faculty members run these programmes. The records was used to describe the number of medical postgraduate students in each year, graduating from the programmes being offered at UNZASOM.

### (3) Medical records for international patient referrals at the Ministry of Health (MOH)

Patients in Zambia requiring specialist treatment unavailable in the country have the option of either making private arrangements for referral to international medical facilities or seeking assistance from the government through MOH. As the majority of patients cannot afford private care, many rely on the MOH option. We collected the total number of patients who were on the waiting list and those who had received specialist treatment through MOH at various international medical facilities. Information was collected for 2013 to September 2021, inclusive.

Ethics clearance for this study was obtained from the University of Zambia Biomedical Research Ethics Committee ref number 920–2020. Collected data was coded and entered into Stata, Corp College Station TX, and version 15. The results were summarised using percentages and graphs were prepared using Microsoft Excel work package.

## RESULTS

### Reports from the stakeholder engagements under SHEPIZ

From the workshops conducted, it was gathered that in Zambia, UNZASOM was the leading institution offering medical specialization programmes and its graduates had been instrumental in fostering improvement of medical training in many institutions around the country. Table 1 gives a summary of the stakeholders who were engged and a summary of the programmes that they recommened as currently feasible at UNZASOM.

Information was also gathered that the first postgraduate programmes introduced at UNZASOM were Masters of Medicine in Paediatrics and Child Health, Obstetrics and Gynecology, Internal Medicine and General surgery with the first graduates in 1986. Programmes introduced in later years factored in some subspecializations including Orthopaedics and Trauma Surgery, Urology, Anaesthesia and Intensive Care, Paediatric Surgery, Ophthalmology, Gastroenterology, Pathology, Infectious Diseases, Neurology and Psychiatry. Table 2 summarises these programmes, showing the years for first graduates.

With the stakeholder engagement, it was clear that these programmes were insufficient and required improvements. The programmes had scored several achievements producing a pool of specialists that are now leaders in their fields with a high retention rate.

With the information gathered, a total of thirteen programmes were subsequently prepared and approved by the University of Zambia (Table 3). These are yet to be reviewed by Higher Education Authority (HEA). With the exception of the Dermatology and Venereology which will be a Masters in Medicine programme, the other twelve will offer subspecialist qualifications. The duration of the programmes will be two years and the degrees awarded will be Master of Science in Specialized Medicine (MSc).Gynaecological Oncology programme will be combined a MMed and subspecialist course, Targeted trainees will be qualified medical subspecialists in respective fields as indicated in table 3.

### Medical specialists trained at University of Zambia School of Medicine (UNZASOM)

For the above proposed programmes to be successful, there was need to have a pool of specialists with a background training that would make subspecialization possible. From the first graduates in 1986 up to 2019, UNZASOM produced 351 medical specialists, 63 (18%) in Internal Medicine, 77 (22%) in Obstetrics &Gynaecology, 82 (23%) in Paediatrics&Child Health, 68 (19%) in General Surgery, 17 (5%) in Anaesthesia & Critical Care, 20 (6%) in Orthopaedics and Trauma and 8 (2%) in Urology. Others were in Ophthalmology 3 (1%), Psychiatry 4 (1%), Infectious Diseases 3 (1%) and Paediatric Surgery contributing 2 (1%) and Pathology 4 (1%).

The number of graduates each year was fluctuating with noticeable reductions in 1987, 1989 and 1991, Figure 1. The highest number of was in 2016 with 47graduates, with the highest numbers being in Internal Medicine (n=8), Obstetrics and Gynaecology (n=9) and Paediatrics and Child Health (n=10). Another year with a relatively high number of graduates was 2019 with a total of 41, with the largest numbers being from Obstetrics &Gynaecology (n=10) and General Surgery (n=8).

### An overview of patients on the Ministry of Health (MOH) register requiring subspecialist treatment outside Zambia

Records at MOH showed that since 2013, a total of 920 patients had requested MOH assistance to facilitate their referral for subspecialist medical care outside Zambia. Of these, 407 (44%) were sent for treatment, with the remainder either still on the waiting list or having had bad outcomes. The years 2016, 2020 and 2021 saw the lowest proportion of patients send abroad, Figure 2.

## DISCUSSION

The need for enhancement of medical subspecialists training programmes is evident in many parts of Africa. It requires regular collection and analysis of data from medical training institutions within the continent.^10^ Due to limited opportunities, many Africans seek medical specialist training from overseas institutions in high income countries.^11^In this study, we collected information of the status of medical specialization and subspecializing in Zambia, including an analysis of current achievements. We found evidence that a number of specialists had been trained in Zambia but with very limited numbers of subspecialists, fueling the demand for medical tourism.

Prior to initiating the SHEPIZ programme, gaps in clinical training in Zambia were identified and innovative approaches were planned to develop clinical training skills of already trained specialists at UNZASOM. Collaborative partnerships in medical specialty training have the potential to improve training opportunities in sub-Saharan Africa.^12^Specialist programmes that were introduced over thirty years ago have scored considerable successes. Despite the limited opportunities for subspecialisation, some Zambian specialists managed to pursue them abroad and return to contribute toward provision of these programmes at UNZASOM. The twelve subspecialist programmes that have been developed were carefully selected and it is hoped that in a few years, more will be added on. These programmes will target existing specialists in Zambia and some neighbouring countries, providing a platform for affordable, easily accessible and locally relevant subspecialist training. Within the SHEPIZ project, there is provision to support Zambian specialists at UNZASOM or UTH to go for 3–6 month attachments in centres providing subspecialist techniques that are not locally available. These attachments are available even to faculty members who might require specific additional training or clinical practice involving modern techniques. The majority of these trainings would be regional, taking advantage of already established South to South collaborations. Memoranda of understanding will be agreed upon this such institutions to facilitate placement of trainees.

Another approach would be to organizemulti-centre training programmes to utilize resources in various centres in the region. One such example is the Emergency Medicine training programme involving Botswana and South Africa (SA).^7^ In this initiative, The University of Botswana (UB) partnered with established Emergency Medicine postgraduate program in SA. The UB curriculum was established based on SA academic, clinical and research requirements. As part of this arrangement, the SA written examination was accredited to be taken under supervision in Botswana and some clinical rotations were also arranged to be taken in SA.^7^In Ethiopia, the East African Training Initiative engaged expert volunteer faculty from Europe and the USA to establish high level subspecialist medical training.^13^

World over, there are variations in terminology and classification of medical specialties and subspecialties. In Zambia, individuals with MMed or equivalent qualifications are registered as medical specialists by the Health Professions Council of Zambia (HPCZ), a statutory regulatory body responsible for the registration, licensing and accreditation of health practitioners, centres and training programmes.^14^ Currently, there is no separate register for subspecialists and the medical qualification structure. The Zambia Qualifications Authority (ZAQA) provides guidance for placement of qualifications and further provides descriptors of the same. These new sub-specialist programmes will therefore, need to be incorporated into this framework. Registration of the qualifications is done by the Higher Education Authority (HEA) but currently there is no clear framework for these new programmes. This compromises the incentive to subspecialise and many local employers do not provide extra enumeration for subspecialisation. The need to reorganize medical subspecialisation and qualification structure in Zambia is therefore, an urgent one. As part of the SHEPIZ project, stakeholders from HPCZ, HEA and ZAQA have been engaged to revise hierarchical structure of medical graduates. the qualification structure for medicine in Zambia. Special recognition of specialists associated with improved incentives could contribute to a reduction in specialized health worker emigration to better resourced countries.^15^

Graduates from the UNZASOM have been very instrumental in fostering improved medical care and training in Zambia. Over the years, there has been other projects that contributed to enhancing medical education at UNZASOM. Notable examples are the Medical Education Partnership Initiative (MEPI)^16,17^ and Zambia Educational Partnership for Advance Care and Training (ZEPACT) that provided a firm foundation for SHEPIZ initiatives. Major themes in MEPI and ZEPACT programmes included clinical competencies training and assessment including simulations, use of clinical protocols with audits of related outcomes and various models for training on recent advancements in patient management.

Global disparities in medical technologies, skilled personnel and economic dynamics are some of the major drivers of medical tourism.^18^ Medical tourism is indeed beneficial in specific cases, but at community and country level, the benefits are usually restricted to a select few. In Zambia, the majority of patients needing subspecialist care abroad never get the opportunity due to resource limitations. Our analysis has demonstrated the need for an alternative strategy such as training subspecialists locally which could be employed as a more cost effective and equitable approach.

## LIMITATIONS

Our study had some limitations. Firstly, we were unable to get cost estimates for sending patients abroad, as this would have given a clearer indication of how training specialists locally would be cost effective. Secondly, we did not have information on the reasons for the international referrals. Third, information on the success rates of students enrolled in postgraduate programmes at UNZASOM was also not available to us. Despite the limitations, we have comprehensively summarised the situation pertaining to medical subspecialisation training in Zambia, experiences that could also be used in other sub-Saharan Africa countries as well.

## CONCLUSION

Medical subspecialisation in Zambia is limited and efforts have been put in place to contribute towards improving the situation and reducing on the resources being used for medical tourism.

## Figures and Tables

**Table 1:** Stakeholders that were engaged to collect information on the need and capacity of sub-specialist training at UNZASOM

Department	Level of specialisation	Number	Sub-specialities training that could be started at UNZASOM
Paediatrics and Child Health	Consultant Paediatricians	2	1. Gastroenterology 2. Infectious Diseases
Internal Medicine	Consultant Physicians	6	1. Gastroenterology 2. Nephrology 3. Dermatology
	MMed trainee	1	
Pathology	Consultant pathologists	2	1. Forensic pathology
	MMed trainee	1	
Surgery	Consultant surgeons	8	1. Breast surgery 2. Urology 3. Various ophthalmology subspecialist courses
Obstetrics and Gynaecology	Consultant obstetricians	4	1. Gynaecological oncology
Medical Education and Training	Trained consultants	4	1. Guided the formulation of all the programmes

**Table 2:** Clinical postgraduate programmes being offered at the University of Zambia School of Medicine

Department	Programme	Duration of programme	Year of the first graduate
Surgery	MMed General Surgery	4 years	1986
	MMedOrthopaedics and Trauma Surgery	4 years	2000
	MMed Urology	4 years	2012
	MMedAnaesthesia and Intensive Care	4 years	2015
	MMedPaediatric Surgery	4 years	2019
	MMed Ophthalmology	4 years	2016
Internal Medicine	MMed General Internal Medicine	4 years	1986
	MMed Internal Medicine and Gastroenterology	5 years	2021
	MMed Internal Medicine and Infectious Diseases	5 years	2017
	MMed Internal Medicine and Neurology	5 years	2020
Paediatrics	MMedPaediatrics and Child health	4 years	1986
Obstetrics and Gynaecology	MMed Obstetrics and Gynaecology	4 years	1988
Psychiatry	MMed Psychiatry	4 years	2015
Pathology	MMed Pathology	4 years	2016

**Table 3:** New medical specialist programmes to be offered at UNZASOM

Programmes	Designation	Duration of course	Pre-requisite qualification
Department of Surgery			
1. Paediatric anaesthesia	Sub-specialty	2 years	MMed General anaesthesia
2. Breast surgery	Sub-specialty	2 years	MMed General surgery
3. Urology	Sub-specialty	2 years	MMed General surgery
4. Glaucoma	Sub-specialty	2 years	MMed Ophthalmology
5. Vitreo-retinal surgery	Sub-specialty	2 years	MMed Ophthalmology
6. Paediatric Ophthalmology and Strabismus Surgery	Sub-specialty	2 years	MMed Ophthalmology
7. Community eye health	Sub-specialty	2 years	MMed Ophthalmology
Department of Internal Medicine			
8. Adult gastroenterology	Sub-specialty	2 years	MMed Internal Medicine
9. Dermatology and Venereology	Specialty	4 years	MBChB
Department of Pathology			
10. Forensic pathology	Sub-specialty	2 years	MMed Pathology
Department of Obstetrics and Gynecology			
11. Gynaecological Oncology	Sub-specialty	5 years	MMed Obstetrics and Gynaecology
Department of Paediatrics			
12. Paediatric infectious diseases	Sub-specialty	2 years	MMedPaediatrics and Child Health
13. Paediatric gastroenterology	Sub-specialty	2 years	MMedPaediatrics and Child Health

* Pre-requisite course are as indicated or their equivalent. MMed is Master of Medicine while MBChB is Bachelor of Medicine and Surgery

## What is already known on this topic:

- There is a shortage of medical specialists in many African countries including Zambia
- Some specialist programmes were introduced at the University of Zambia School of Medicine in 1986 but without a focus on subspecialties
- There is a heavy reliance on medical tourism for patients needing subspecialist care

## What this study adds:

- The University of Zambia School of Medicine has capacity to run selected medical subspecialty programmes
- More than half of patients requiring subspecialist care do not get it in good time, with long waiting lists
- Introduction of subspecialty programmes in Zambia could contribute towards a reduction in the demand for international medical consultations

## References

[1] Cox M, Masunge J, Geduld H. A successful hybrid emergency medicine postgraduate partnership in Southern Africa. Afr J Emerg Med. 2020;10(Suppl 1):S56–S59. 33318903 10.1016/j.afjem.2020.02.005 PMC7723910

[2] Health Professions Council of Zambia. https://www.hpcz.org.zm. Accessed on 29th September 2021.

[3] Kayamba V, Mutale W, Cassell H, Heimburger DC, Shu XO. Systematic Review of Cancer Research Output From Africa, With Zambia as an Example. JCO Glob Oncol. 2021;7:802–810. 34077269 10.1200/GO.21.00079 PMC8459799

[4] Kiguli-Malwadde E, Talib ZM, Wohltjen H, Connors SC, Gandari J, Banda SS, Maggio LA, van Schalkwyk SC. Medical education departments: a study of four medical schools in Sub-Saharan Africa. BMC Med Educ. 2015;15:109. 26126821 10.1186/s12909-015-0398-y PMC4486688

[5] Michelo C, Zulu JM, Simuyemba M, Andrews B, Katubulushi M, Chi B, Njelesani E, Vwalika B, Bowa K, Maimbolwa M, , Strengthening and expanding the capacity of health worker education in Zambia. Pan Afr Med J. 2017;27:92. 28819513 10.11604/pamj.2017.27.92.6860 PMC5554665

[6] Mogaka JJO, Mupara L, Tsoka-Gwegweni JM. Ethical issues associated with medical tourism in Africa. J Mark Access Health Policy. 2017;5(1):1309770. 28740618 10.1080/20016689.2017.1309770 PMC5508395

[7] Morel T, Maher D, Nyirenda T, Olesen OF. Strengthening health research capacity in sub-Saharan Africa: mapping the 2012–2017 landscape of externally funded international postgraduate training at institutions in the region. Global Health. 2018;14(1):77. 30064479 10.1186/s12992-018-0395-0 PMC6066939

[8] Muula AS. Specialist training programs for African physicians. Croat Med J. 2006 Oct;47(5):789–91. 17042072 PMC2080465

[9] Mwanza KE, Stassen W, Pigoga JL, Wallis LA. The views and experiences of Zambia’s emergency medicine registrars in South Africa: Lessons for the development of emergency care in Zambia. Afr J Emerg Med. 2021;11(1):65–69. 33680723 10.1016/j.afjem.2020.08.002 PMC7910180

[10] Ndlovu N, Ndarukwa S, Nyamhunga A, Musiwa-Mba P, Nyakabau AM, Kadzatsa W, Mushonga M. Education and training of clinical oncologists-experience from a low-resource setting in Zimbabwe. Ecancermedicalscience. 2021;15:1208. 33912233 10.3332/ecancer.2021.1208 PMC8057777

[11] Nwadiuko J, Switzer GE, Stern J, Day C, Paina L. South African physician emigration and return migration, 1991–2017: a trend analysis. Health Policy Plan. 2021; 29:czaa193. 10.1093/heapol/czaa193 33778873

[12] Omaswa F, Kiguli-Malwadde E, Donkor P, Hakim J, Derbew M, Baird S, Frehywot S, Gachuno OW, Kamiza S, Kibwage IO, ,The Medical Education Partnership Initiative (MEPI): Innovations and Lessons for Health Professions Training and Research in Africa. Ann Glob Health. 2018; 84(1):160–169. 30873813 10.29024/aogh.8 PMC6586907

[13] Schluger NW, Sherman CB, Binegdie A, Gebremariam T, Kebede D, Worku A, Carter EJ, Brändli O. Creating a specialist physician workforce in low-resource settings: reflections and lessons learnt from the East African Training Initiative. BMJ Glob Health. 2018;3(5):e001041. 10.1136/bmjgh-2018-001041 PMC6144898 30245867

[14] Simuyemba M, Talib Z, Michelo C, Mutale W, Zulu J, Andrews B, Nzala S, Katubulushi M, Njelesani E, Bowa K, Maimbolwa M, Mudenda J, Mulla Y. Strengthening faculty recruitment for health professions training in basic sciences in Zambia. Acad Med. 2014;89(8 Suppl):S98–S101. 25072591 10.1097/ACM.0000000000000352 PMC4115288

[15] Talib Z, Narayan L, Harrod T. Postgraduate Medical Education in Sub-Saharan Africa: A Scoping Review Spanning 26 Years and Lessons Learned. J Grad Med Educ. 2019;11(4 Suppl):34–46. 31428258 10.4300/JGME-D-19-00170 PMC6697307

[16] The Ultimate List of Medical specialties and subspecialties. https://www.sgu.edu/blog/medical/ultimate-list-of-medical-specialties/. Accessed on 28th September 2021.

[17] University of Zambia website, https://www.unza.zm/schools/medicine/about, accessed on 4th August 2021.

[18] World Health Organization: Zambia steps survey for non communicable diseases. Zambia report for 2017. https://www.afro.who.int/publications/zambia-steps-survey-non-communicable-diseases-zambia-report-2017, Accessed on 28th September 2021.

